# Optimizing Maxillary Aesthetics of a Severe Compromised Tooth through Orthodontic Movement and Dental Implants

**DOI:** 10.1155/2014/103808

**Published:** 2014-01-12

**Authors:** Rafael Scaf de Molon, Erica Dorigatti de Avila, Joni Augusto Cirelli, Mauricio de Almeida Cardoso, Leopoldino Capelozza-Filho, Luiz Antonio Borelli Barros

**Affiliations:** ^1^Department of Diagnosis and Surgery, School of Dentistry at Araraquara, Universidade Estadual Paulista (UNESP), Rua Humaitá 1680, 14801-903 Araraquara, SP, Brazil; ^2^Department of Dental Materials and Prosthodontics, School of Dentistry at Araraquara, Universidade Estadual Paulista (UNESP), Rua Humaitá 1680, 14801-903 Araraquara, SP, Brazil; ^3^Discipline of Orthodontics, University of Sagrado Coração, Rua Irmã Arminda 10-50, 17011-160 Bauru, SP, Brazil; ^4^Department of Social Dentistry, School of Dentistry at Araraquara, Universidade Estadual Paulista (UNESP), Rua Humaitá 1680, 14801-903 Araraquara, SP, Brazil

## Abstract

Treatment of severe compromised tooth in the maxillary anterior area still poses great challenge to the clinicians. Several treatment modalities have been proposed to restore the function and aesthetics in teeth with advanced periodontal disease. The present study aims to report a case of traumatic injury of a left-maxillary central incisor with ridge preservation, orthodontic movement, and implant therapy. A 45-year-old woman underwent the proposed treatment for her left central incisor: basic periodontal therapy, xenogenous bone graft, and guided bone regeneration (GBR). Six months after the graft procedure, orthodontic movement by means of alignment and leveling was made and a coronal displacement of the gingival margin and vertical bone apposition could be observed after 13 months of active movement. Afterwards, a dental implant was placed followed by a connective tissue graft and immediate provisionalization of the crown. In conclusion, orthodontic movement was effective to improve the gingival tissue and alveolar bone prior to implant placement favoring the aesthetic results. Six years postoperatively, the results revealed height and width alveolar bone gain indicating that the treatment proposed was able to restore all the functional and aesthetic parameters.

## 1. Introduction

Single implant therapy is a predictable treatment and has high success rates, at least when adequate bone volume is present. However, severe compromised tooth in the maxillary aesthetic region poses a great challenge to implant therapy. A correct diagnosis, absence of systemic conditions such as diabetes mellitus [1], an adequate treatment plan, improvement of surgical techniques, and multidisciplinary team planning play an important role in the success of complex cases [2]. According to Savi et al. [3] to achieve an adequate aesthetic result in anterior upper regions with dental implants, favorable periodontal tissue and bone conditions should be present.

There are several treatment options to restore the aesthetic and function of a compromised anterior tooth. Different treatment modalities to hard and soft tissue formation at the site of tooth extraction are used, including forced orthodontic eruption [4, 5], ridge augmentation by means of bone and connective tissue graft [2], guided bone regeneration (GBR), immediate or delayed implant placement, and a combination of those [6]. Implant therapy can be complex due to numerous local anatomic or traumatic factors resulting in aesthetic commitment in the maxilla. These factors involve thin gingival biotype, thin buccal bone wall, bone dehiscence, and absence of soft and hard tissue quality and quantity, which hampers the success of aesthetic outcomes.

After the tooth extraction, a wound healing process occurs after 6 weeks, while the bone fill in the alveolus takes up to 4 months [7]. However, ridge alterations are expected, as demonstrated by previous study [8] where in a randomized clinical trial involving 24 patients requiring molar tooth extraction, the authors showed that the reduction in ridge width and height was 2.6 mm and 0.9 mm, respectively. Therefore, regenerative techniques have been recommended following tooth extraction especially when the buccal wall was lost, to allow ridge augmentation improving soft and hard tissue volume for the time of implant placement [7].

The present study reports a case of traumatic injury of a left-maxillary central incisor where the treatment involved regenerative procedures by means of xenogenous bone graft, GBR, orthodontic movement, implant placement, connective tissue graft, and immediate provisionalization of the crown. Here, we demonstrate for the first time that orthodontic movement can result in coronal displacement of gingival margin and vertical bone apposition favoring better aesthetic outcomes for implant rehabilitation.

## 2. Case Report

**Figure 1:**
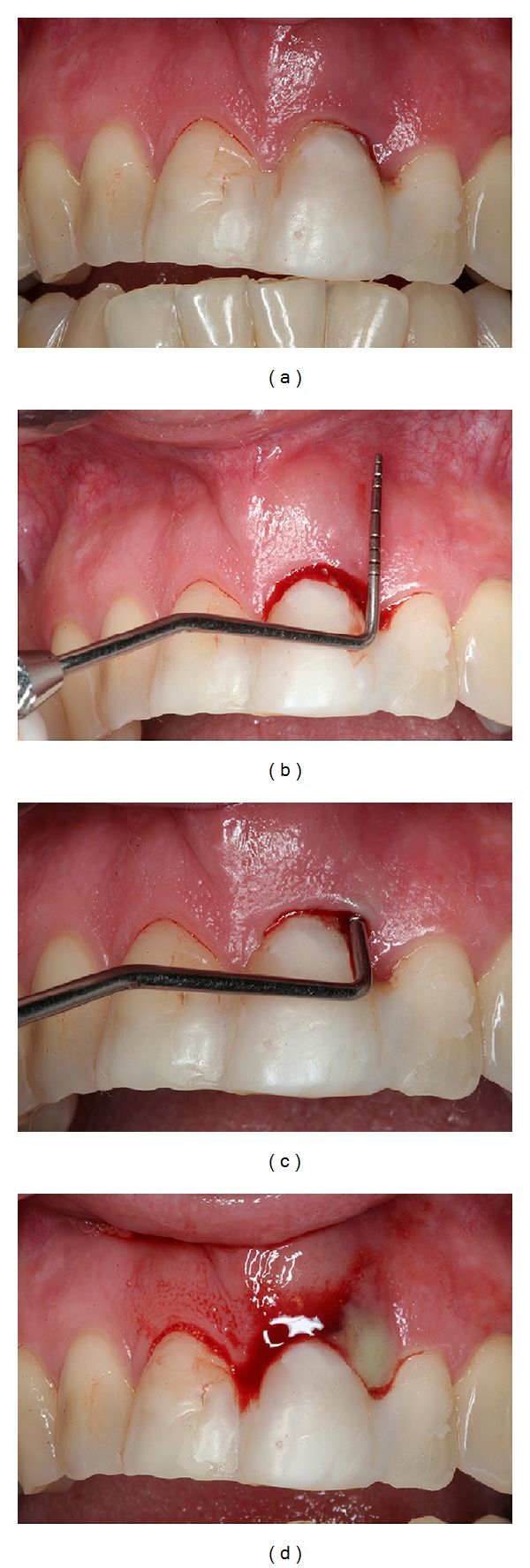
Pretreatment intraoral examination showing extensive periodontal pocket with 11 mm of probing depth, bleeding, and suppuration on probe.

**Figure 2:**
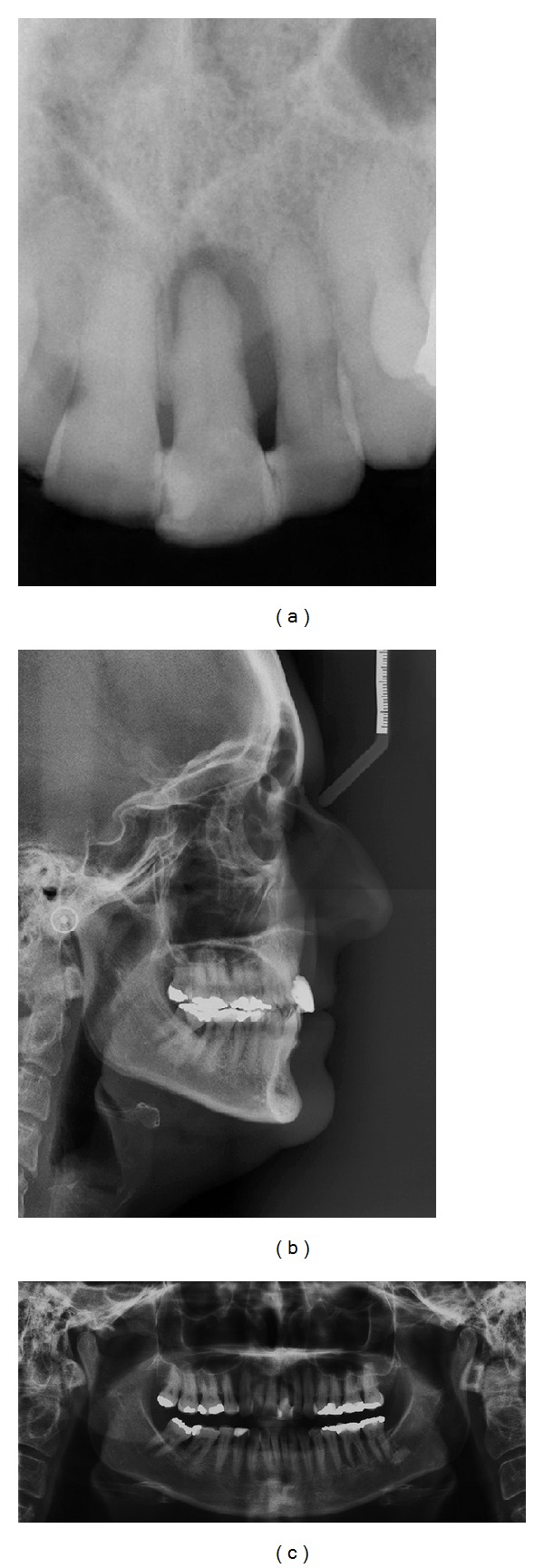
Pretreatment periapical, cephalometric, and panoramic radiographs.

A 45-year-old Caucasian female was referred to the Department of Periodontology, for evaluation and treatment of her maxillary central incisor with the chief complain of pain and swelling. Medical history was not contributory and the patient denied use of alcohol or smoking. Clinical examination revealed poor oral hygiene, localized gingival recessions, and thick gingival tissue. Probing depths ranged from 3 to 5 mm but in her left-maxillary central incisor a localized 11 mm probing depth pocket with spontaneous bleeding and suppuration was detected at the buccal and mesial faces of the tooth (Figure 1). The tooth was extruded and splinted with resin in the adjacent teeth, resulting in absence of mobility. Periapical, cephalometric, and panoramic radiograph showed generalized alveolar bone loss and severe bone resorption and periapical lesion in the left-maxillary central incisor (Figure 2).

Based on clinical and radiographic examinations, tooth extraction followed by reconstructive procedures, orthodontic movement, and implant placement was proposed and accepted by the patient to improve the aesthetics with harmonious occlusion. Written informed consent was obtained prior to initial treatment.

**Figure 3:**
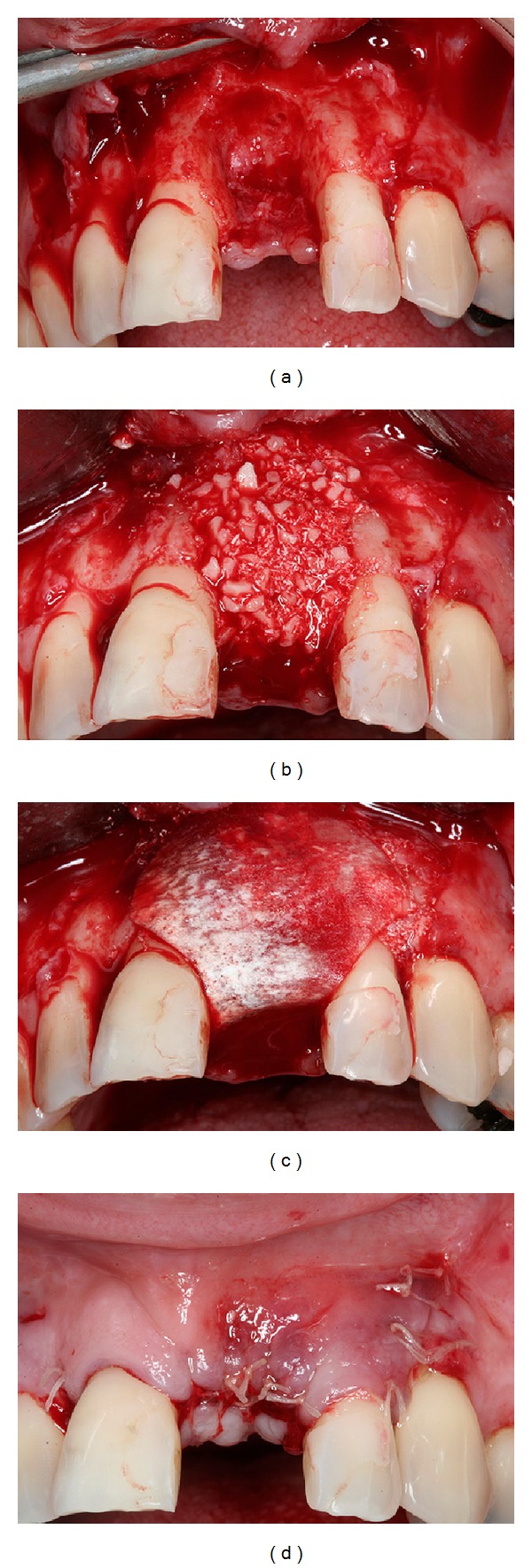
Guided bone regeneration with xenogenous bone graft and membrane barrier followed by a simple suture.

The patient underwent a periodontal treatment involving instructions and reinforcement in her oral hygiene efforts followed by a scaling and root planing in the entire dentition. After one week, a full-thickness flap was elevated on the buccal and lingual aspects from the right canine to the left canine and the left central incisor was extracted. The area was thoroughly debrided and the adjacent teeth were scaled and planed. Immediately afterward, an anorganic bovine bone (Bio-Oss, Geistlich, Wolhusen, Switzerland) was applied into the defect and an absorbable collagen membrane (GenDerm, Genius, Baumer, Sao Paulo, Brazil) was placed over the graft, covering all the defect and adjacent bone borders. The flap was advanced to completely cover the membrane barrier. The 5.0 polytetrafluoroethylene thread (Ethicon, Somerville, NJ, USA) and simple suture technique secured the flap in place (Figure 3). The patient was seen two weeks after surgery for suture removal and provisional resin crown making. Postoperatively visits included oral hygiene instructions and plaque control every month for 6 months after surgery.

**Figure 4:**
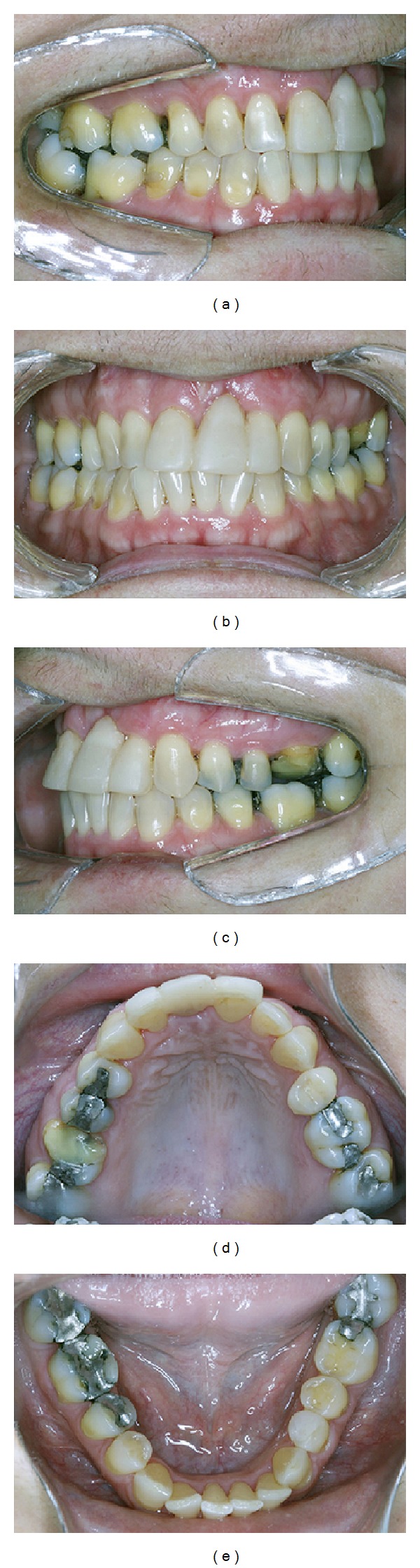
Pretreatment intraoral photographs.

**Figure 5:**
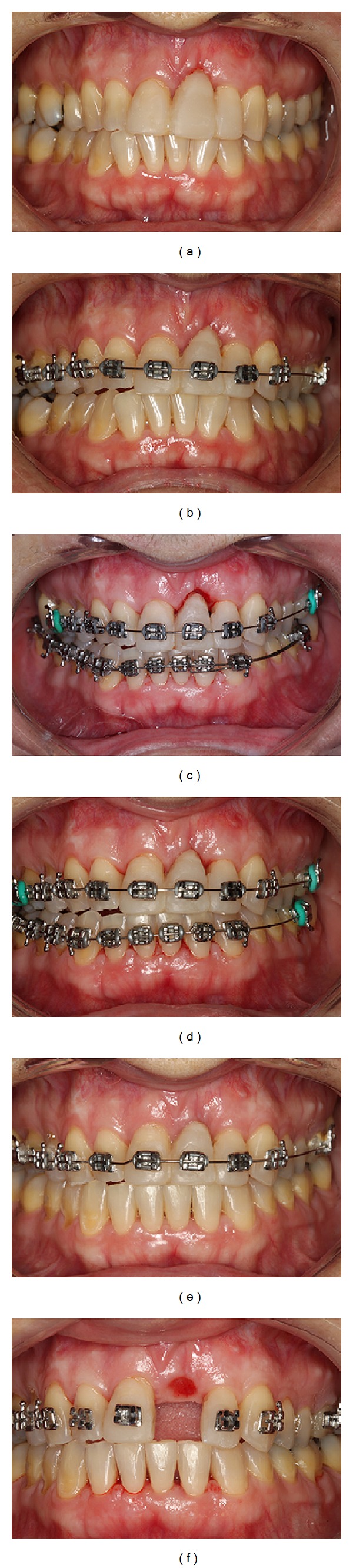
Orthodontic movement progress showing coronal gingival displacement favoring the aesthetic results after 13 months of active orthodontic movement by means of alignment and leveling.

**Figure 6:**
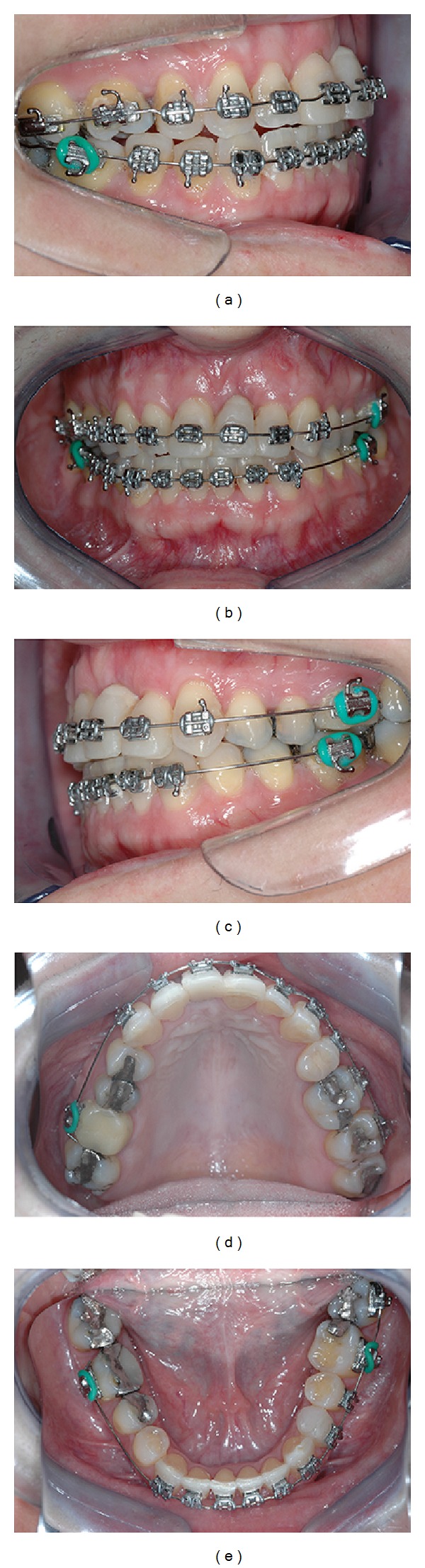
Posttreatment orthodontic movement showing acceptable occlusion.

**Figure 7:**
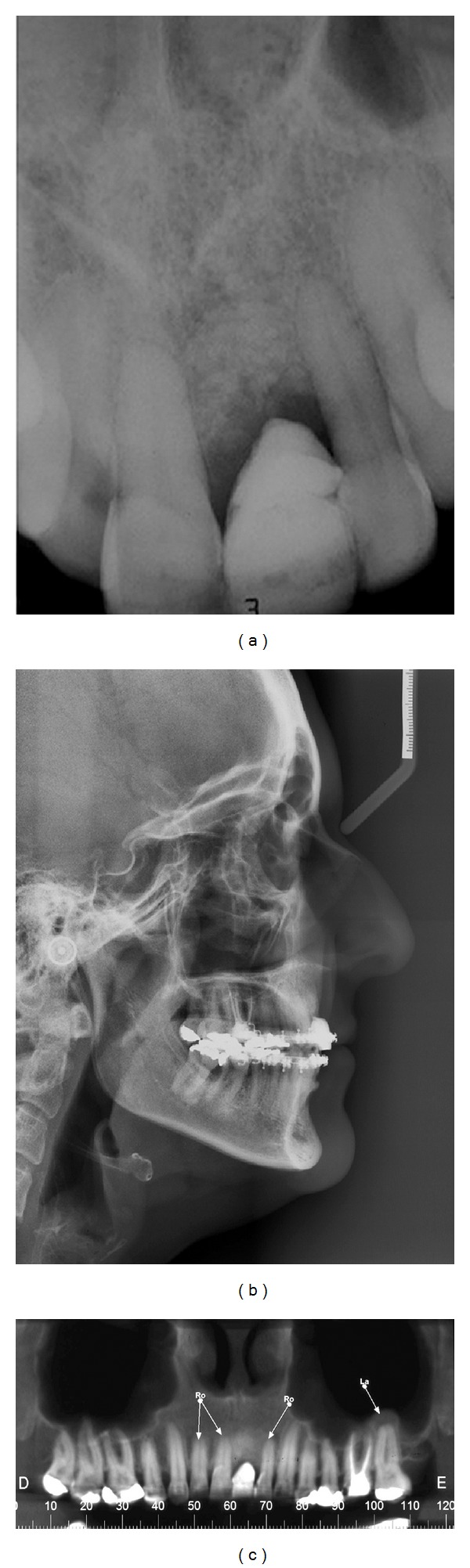
Periapical, cephalometric, and panoramic radiography after orthodontic movement showing alveolar bone augmentation allowing implant placement.

At 6 months, the patient showed good plaque control, and great bone width augmentation, but a vertical gingival defect was presented in the area where the tooth was extracted due to a vertical bone deficiency (Figure 4). She showed a straight profile and a slightly facial asymmetry with history of poor occlusion and acceptable face. At this time, orthodontic treatment was planned to correct the dental malposition by means of alignment and leveling [9]. Brackets were placed on the mandibular and maxillary arch from molar to molar with a steel .018 wire. The bodily movement started to realign the teeth, with sectional wires and light forces. After 13 months of active orthodontic movement an impressive coronal displacement of gingival tissue and bone tissue apposition could be observed, leaving the gingival margin in the same position of the adjacent teeth yielding better aesthetic results in future's surgical and prosthetic procedures (Figure 5). The results of orthodontic treatment could be observed with a harmonious occlusion at the end of orthodontic treatment (Figure 6). Periapical, cephalometric, and panoramic radiography showed great bone volume augmentation allowing implant placement (Figure 7).

**Figure 8:**
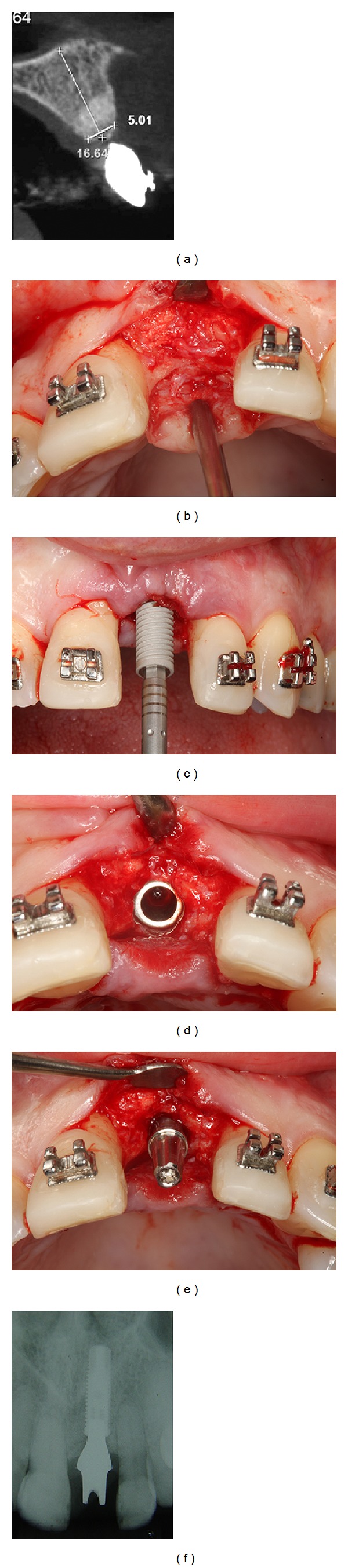
Implant placement after orthodontic movement showing bone volume augmented allowing implant placement in an ideal 3-dimensional position and immediate installation of a provisional abutment.

**Figure 9:**
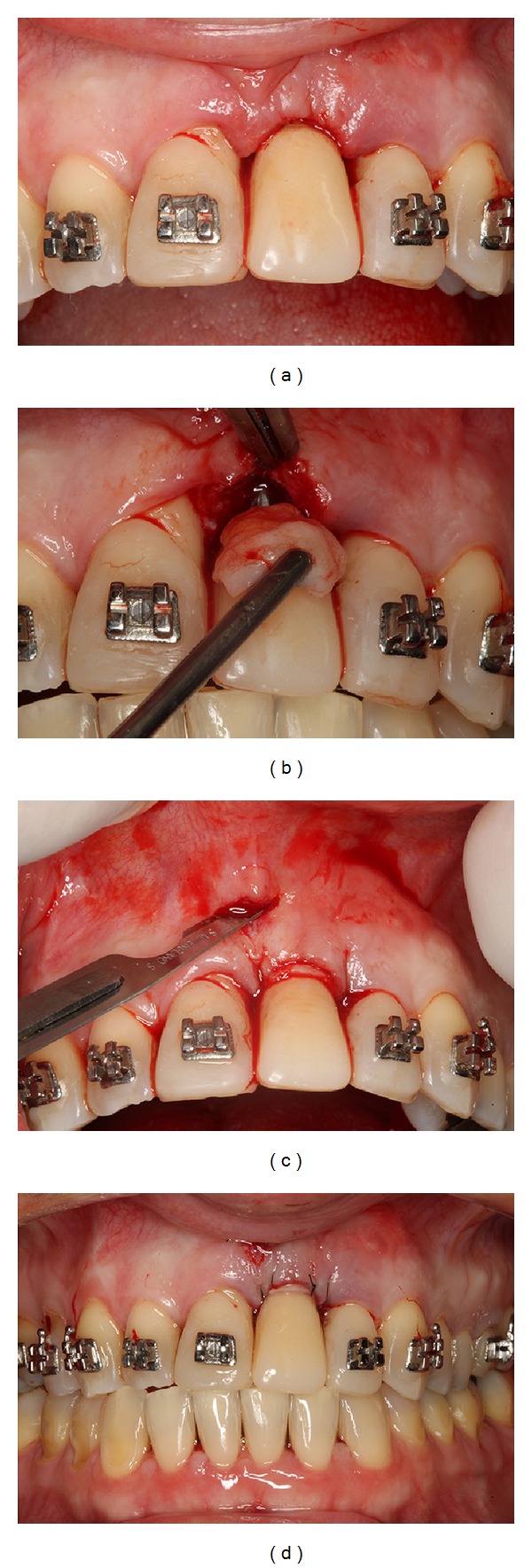
Installation of a provisional crown followed by autogenous connective tissue graft, frenum removal, and simple suture.

**Figure 10:**
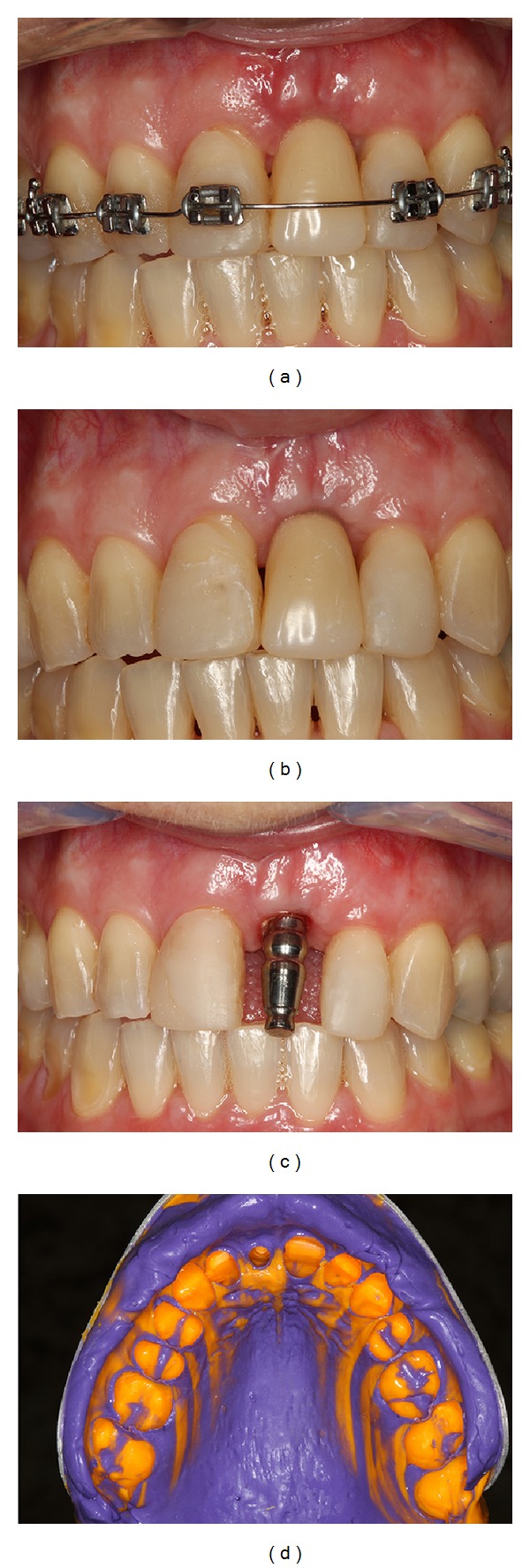
Transfer impression for coping fabrication.

**Figure 11:**
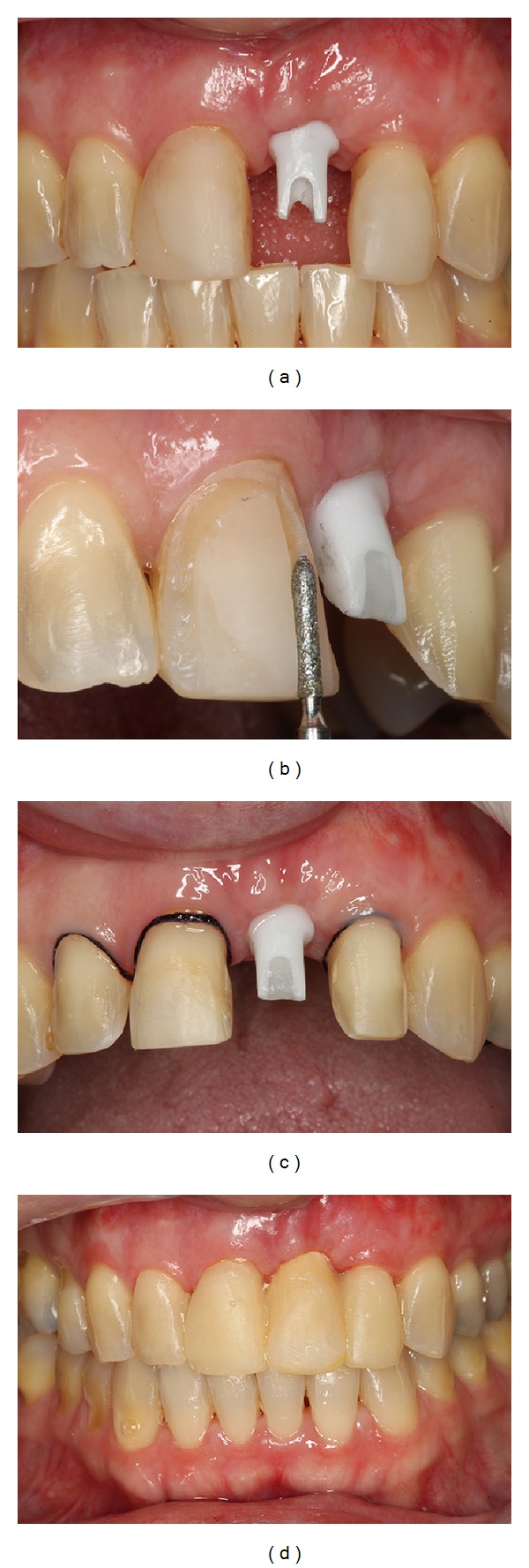
Zirconia custom abutment installed and teeth preparation to receive the final prosthesis.

**Figure 12:**
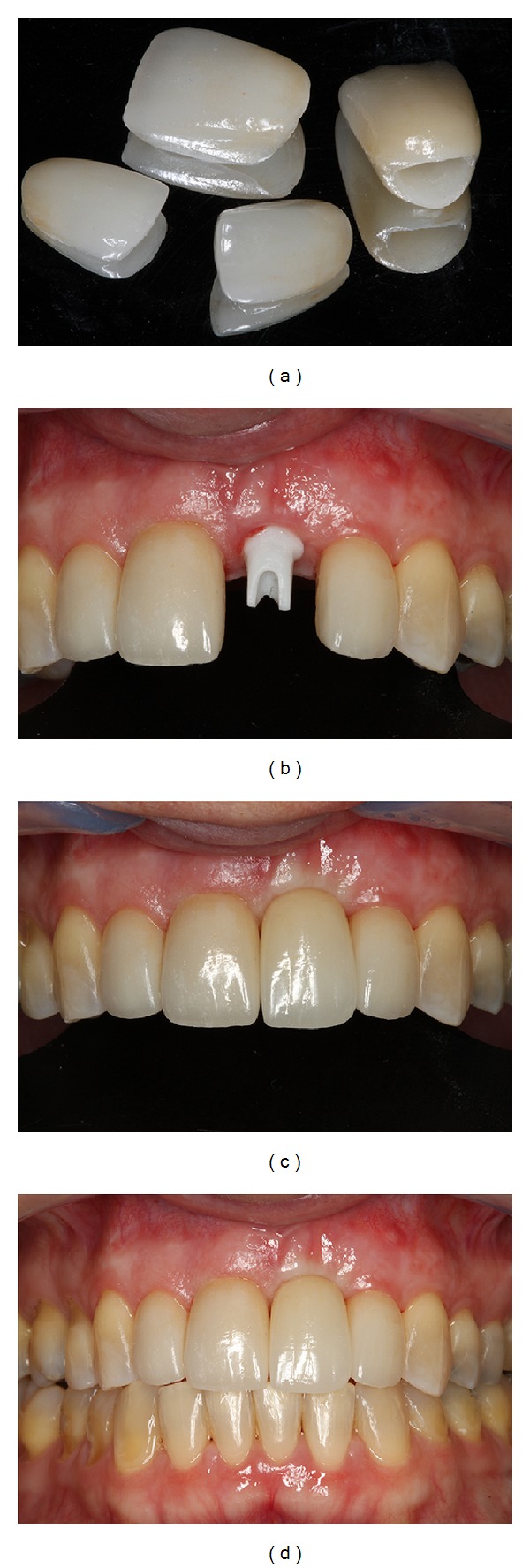
Final prosthesis installed and final result showing excellent aesthetic results.

After orthodontic treatment, a dental implant was planed to restore the aesthetic and function. A minimally invasive surgical technique was made that allows the implant (4.3 × 13 mm Cone Morse, Neodent, Curitiba, PR, Brazil) installation in an ideal 3-dimensional position (Figure 8). Immediately, a provisional abutment and a crown were installed. To improve the soft tissue width around the implant, an autogenous connective tissue graft was placed, the labial frenum was excised, and a simple suture was performed to maintain the graft stable (Figure 9). At 4 months, the prosthetic procedures were started to create a definitive metal-free crown. The transfer impression for coping fabrication was performed (Figure 10) and a zirconia custom abutment was made through the CAD/CAM system (Figure 11). Then, the adjacent teeth were prepared and a feldspathic porcelain (IPS Empress II: lithium-disilicate glass-ceramic restoration, Ivoclar, Vivadent) crown was prepared and installed over the zirconia abutment and the prepared teeth allowing excellent aesthetic results (Figure 12).

**Figure 13:**
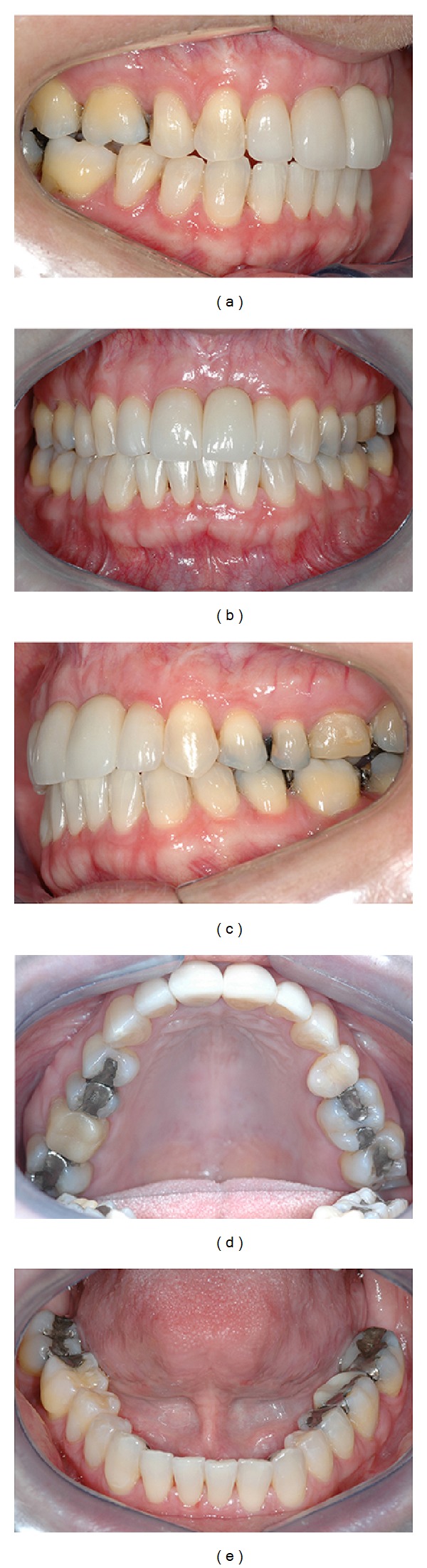
6-year follow-up intraoral photographs.

**Figure 14:**
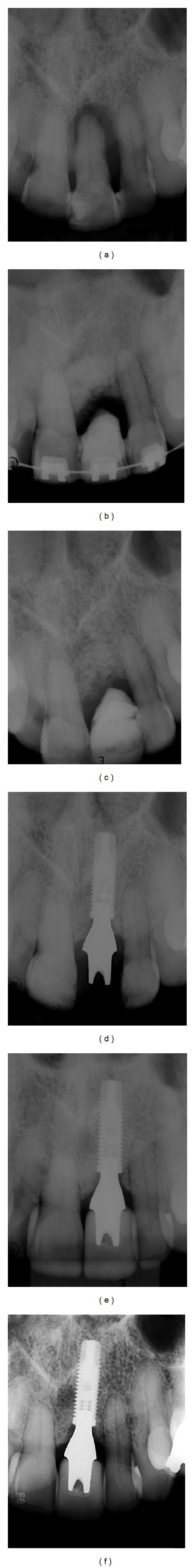
6-year follow-up periapical radiographies. (a) Initially; (b) after bone grafting and GBR and beginning of orthodontic treatment; (c) after orthodontic treatment; (d) immediate implant placement; (e) after prosthesis installation; (f) 6 years postoperatively.

Six years postoperatively, clinical examination showed absence of gingival recession, no probing depths, and no bleeding on probing or suppuration. Patient's smile esthetics was improved and a satisfying occlusion was achieved (Figure 13). Periapical radiographies evaluation revealed height and width alveolar bone gain, especially the vertical bone apposition, indicating that the treatment proposed was able to restore all the functional and aesthetic parameters (Figure 14).

## 3. Discussion

This clinical case adds to the growing evidence that reconstructive surgical procedures combined with orthodontic movement by means of alignment and leveling reconstructed the lost periodontal tissues making the final prosthetic rehabilitation easier to be achieved. The interesting clinical finding noted in this case is that the orthodontic movement led to a coronal displacement of the gingival tissue and bone vertical apposition resulting in the soft tissue margin in the same level of the adjacent teeth avoiding discrepancies in the size of the clinical crown with relation to adjacent teeth.

Periodontal disease (PD) is a chronic inflammatory condition that results in clinical attachment loss, pocket formation, and alveolar bone resorption [10, 11]. When a trauma affects a tooth, prosthetic rehabilitation may be worsened in consequence of extensive bone dehiscences and fenestrations around the tooth from preexisting periodontal disease and/or periapical lesion. This kind of defect usually needs reconstructive procedures to restore the original anatomy of the lost periodontal tissues. This situation turns the immediate implant placement in an ideal 3-dimensional position unfeasible due to the absence of bone tissue and undesirable morphological changes after tooth extraction that can reach 50% in ridge width reduction [12]. In the present case, we choose ridge preservation, which is a clinical procedure performed at time of tooth extraction and involves placing a bone graft material into the alveolar socket immediately after tooth removal [13]. This decision was based on a recent systematic review [14], where the authors showed that delayed implants may be at lower risk of implant failure in reconstructed alveolar ridges. Xenogenous bone graft was associated with GBR in order to restore the ridge shape and dimension and to prevent the migration of epithelial and connective cells to the area, limiting the resorptive changes after tooth extraction.

A previous study [9] showed that orthodontic movement can result in coronal displacement of gingival margin that was able to cover a denuded root of a mandibular central incisor. On the other hand, another study [15] showed in rats that orthodontic tooth movement in periodontal bone defects surgically created results in enhanced bone healing and bone apposition. The results of this study corroborate with another study [16], which showed that orthodontic movement has favorable effects on restraining epithelial apical downgrowth and decreasing pocket depth in surgical defects created in rat molar tooth. Here, we demonstrate, for the first time, that this type of movement, by means of alignment and leveling, can result in a coronal gingival displacement and bone vertical apposition even in an edentulous alveolar ridge adjacent to moved teeth. This result allowed the gingival margin to stay in the same level of the adjacent teeth avoiding additional surgical procedures to create an adequate volume of soft tissue, maintaining the clinical crown of the future prosthesis in harmony with the natural teeth and favoring an optimal emergence profile with a provisional crown.

After ridge preservation and orthodontic treatment, we obtained successful clinical results that allowed the implant installation in an ideal 3-dimensional position respecting the implant axis through the cingulum, with the incisal edge slightly lingual and the implant platform 3 mm from the cementoenamel junction of the adjacent teeth. The implant was placed with minimally invasive technique since it has been proposed that great alveolar bone loss occurs after elevation of a mucoperiosteal flap [17]. Soft tissue graft is considered to increase the amount of keratinized gingiva at the same time of implant placement, allowing for predictable and maintainable long-term aesthetic and functional outcomes.

In this case, the initial implant stability over 45 Ncm allowed the immediate provisionalization of the crown, since previous study [18] has proposed that primary stability greater than 30 Ncm is essential to the success of immediate implant provisionalization [19]. The benefits of immediate installation of a provisional crown are optimal gingival contour before definitive prosthesis, shortened treatment time, patient satisfaction, and fewer surgical interventions [6].

In conclusion, severe alveolar bone resorption in consequence of a trauma associated with periodontal disease, ridge preservation for minimizing vertical and horizontal bone resorption after tooth extraction, orthodontic movement to create an acceptable position of the gingival margin, immediate implant placement, and immediate provisionalization of the crown were effective to create an excellent clinical aesthetic result.
